# Chronic Pain in Autistic Youth: Clinical Prevalence and Reflections on Tailoring Evidence-Based Interventions from an Interdisciplinary Treatment Team

**DOI:** 10.3390/children11030312

**Published:** 2024-03-06

**Authors:** Gloria T. Han, Holly S. Heavner, Thomas R. Rains, Alan H. Hoang, Amanda L. Stone

**Affiliations:** 1 Department of Anesthesiology, Vanderbilt University Medical Center, Nashville, TN 37232, USA; alan.hoang@vumc.org (A.H.H.); amanda.l.stone@vumc.org (A.L.S.); 2 Department of Pediatric Rehabilitation, Monroe Carell Jr. Children’s Hospital at Vanderbilt, Vanderbilt University Medical Center, Nashville, TN 37232, USA; holly.s.heavner@vumc.org (H.S.H.); thomas.r.rains@vumc.org (T.R.R.)

**Keywords:** autism, chronic pain, sensory processing, central sensitization, sensorimotor integration

## Abstract

Though there is growing awareness of the overrepresentation of autistic patients in chronic pain clinics, potential adaptations for the assessment and treatment of chronic pain in this population have not yet been established. To address this gap, a retrospective review of electronic medical records and discussions by an interdisciplinary pain treatment team were summarized to inform potential biopsychosocial factors affecting the presentation, assessment, and treatment of chronic pain in autistic youth. Our sample included a record review of 95 patients receiving treatment in an interdisciplinary outpatient pediatric pain clinic. Results indicated that 9% (*n* = 9) of the patients presented to the clinic with a prior diagnosis of autism, but an additional 21% (*n* = 20) were identified as likely meeting criteria for autism based on the clinical assessment of the developmental history, behaviors observed during the clinical encounter(s), and expert clinical judgment, suggesting that the prevalence rate of autism may be closer to 30% in our outpatient pediatric pain clinic. Over half (52%) of the autistic youth presented to the clinic with widespread pain, 60% identified as female, and 6% identified as gender expansive or transgender. Qualitative insights revealed that most of the autistic patients had co-occurring sensory-processing challenges and difficulty in describing their pain, emotions, and somatic experiences and exhibited cognitive inflexibility and social challenges. We summarize our team’s clinical reflections on how autism-relevant biopsychosocial vulnerability factors may contribute to the experience of pain in autistic youth and propose treatment targets and adaptations for the assessment and treatment of pain in this population. Finally, we recommend the need for interventions focused on sensorimotor integration, especially for autistic youth, and describe how pain clinics may be particularly helpful for identifying and supporting autistic females, for whom the potential role of autism in pain experiences had not been considered until receiving treatment in our clinic.

## 1. Introduction

Autism spectrum disorder (hereafter, “autism”) is a neurodevelopmental condition affecting 1 in 36 children [1] and is characterized by difficulties in social communication and interaction and the presence of restricted interests and repetitive behaviors (RRBs) [2]. Data from a nationally representative sample in the United States (2016–2017 National Survey of Children’s Health) have indicated that 8.2% of non-autistic children experience chronic pain, compared to up to 20% of autistic (please note that we use identity-first language throughout the text to align with preferences of most autistic individuals [3]) children [4]. Though there is a growing acknowledgment of a potential overrepresentation of autistic children in pediatric pain clinics [5,6], there remains a need to evaluate how existing interdisciplinary interventions for chronic pain may be tailored or augmented to meet the unique needs of youth with neurodevelopmental and distinct sensory-processing profiles. 

Apparently higher rates of chronic pain among autistic youth suggest that shared biopsychosocial factors may explain the development and prevalence of chronic pain in this population, yet empirical exploration of shared mechanisms is just emerging. Several biopsychosocial factors linking core autism features to co-occurring chronic pain symptoms warrant investigation and consideration in clinical contexts. From a neurobiological perspective, approximately 90% of autistic children have altered sensory experiences [7], including both hyper- and hypo-reactivity to sensory inputs or unusual interest in sensory aspects of the environment (e.g., apparent indifference to pain/temperature, aversions to specific sounds or textures, atypical preferences for smelling or touching objects, and visual fascination with lights or movements) [2]. Atypical sensory profiles have been linked to worse adaptive functioning for autistic youth [8,9], and sensory-processing sensitivity, a genetically influenced trait characterized by a lower sensory threshold, has also been associated with a worse quality of life in adolescents with chronic pain [10]. Sensory sensitivities in autism may also mirror hyperalgesia and general nervous system dysregulation observed in chronic pain patients. Relatedly, medical comorbidities are also common among autistic youth, including connective tissue and joint hypermobility disorders, gastrointestinal distress, and neurological conditions [11] that are associated with pain, indicating the potentiation of physiological pain pathways among autistic youth. Specifically, the high rate of co-occurring “central sensitivity syndromes” (e.g., chronic fatigue, amplified pain, migraines, and irritable bowel syndrome) among autistic individuals suggests the presence of “central sensitization” or augmented sensory signaling of the central nervous system, which may also explain vulnerability for chronic pain [12,13]. 

In addition to physiological processes, autistic youth exhibit psychological characteristics that may modulate the effects of the inhibitory pathways that help to reduce the subjective experience of pain. For example, autistic individuals exhibit difficulties with interoception, the process for sensing internal body sensations [14,15]. Systematic reviews have demonstrated a connection between difficulties with interoceptive accuracy (i.e., noticing and detecting internal body sensations) and chronic pain severity [16]. Autistic individuals also exhibit difficulties with emotional identification and regulation (i.e., alexithymia) [17] and cognitive and behavioral inflexibility [18], additional psychological factors shown to exacerbate chronic pain [19,20,21,22] by hindering the modulation of physiological pain pathways and acquisition of adaptive pain-coping strategies. These psychological features are also thought to explain high rates of depression and anxiety among autistic individuals [23], reflecting a psychological profile that increases vulnerability for chronic pain. 

Social and environmental factors may also predispose autistic individuals to chronic pain. Considering that autism is a condition marked by difficulties with social communication and interaction, autistic individuals experience significant social challenges and persistent relationship stressors that lead to elevated allostatic loads [24] and greater levels of social isolation and loneliness that may further contribute to the emergence and maintenance of chronic pain [25,26]. Further, it is plausible that the presence of chronic pain as a stressor may exacerbate features of autism, increasing the overall burden to health and wellbeing for autistic youth. 

Considering the growing awareness of autistic patients presenting at pediatric pain clinics [27], there is an urgent need for disseminating theoretically supported ways to provide care for patients with autism and chronic pain. Interdisciplinary care models that involve collaboration between pain physicians, psychologists, physical therapists, and occupational therapists show promise for treating pain from a biopsychosocial perspective [28,29], but potential adaptations to better serve autistic patients in pain clinics have not yet been explored or established. Prior work focusing on adapting interventions for autistic youth has primarily focused on modifications to cognitive behavioral therapy (CBT) for treating anxiety and depression [30,31]. Common adaptations include using additional communication strategies (e.g., visuals) to explain psychological concepts, prioritizing structures and routines, accommodating sensory sensitivities in the therapy environment, and allowing for longer sessions and durations of treatment to establish rapport and facilitate learning and information processing. Additional adaptations to better serve the needs of autistic youth may be required in the context of interdisciplinary chronic pain intervention. 

The current study sought to evaluate the prevalence and clinical characteristics of autistic youth presenting to an outpatient interdisciplinary pediatric chronic pain clinic. As there is a dearth of current research to guide treatment recommendations for autistic youth with chronic pain, we provide a clinical commentary on how features of autism affected the assessment of pain and implementation of existing evidence-based interventions based on electronic medical records and team-based discussions over a year of clinical service provision. Guided by a biopsychosocial conceptualization of chronic pain and its treatment, we organize our clinical reflections accordingly and aim to provide recommendations for future clinical research and practice to reduce the negative impacts of chronic pain on autistic youth. 

## 2. Materials and Methods

### 2.1. Setting 

The outpatient pediatric pain service is considered as a component of Monroe Carell Jr. Children’s Hospital at Vanderbilt, a large, urban pediatric medical center in the southeastern United States. In September 2022, we launched a new pediatric pain clinic with an interdisciplinary care model focused on functional restoration for school-aged youth experiencing significant pain-related impairment. This team comprised pain medicine, pain psychology, physical therapy (PT), and occupational therapy (OT), which are considered as the ideal co-treatment environment for children who are significantly functionally disabled owing to chronic pain [32]. 

### 2.2. Clinical Team Characteristics and Roles

The training background and prior experiences of the treatment team members significantly shape the way in which we interact with patients, interpret our experiences, and approach clinical decision-making. Thus, we believe it is important to state that observations provided are reflective of our own personal and professional lived experiences. The pediatric pain physician on our team completed residencies in both general pediatrics and anesthesiology, followed by a board-certified pain medicine fellowship, which included training across both pediatric and adult pain care. The pediatric psychologists on our team both completed doctoral degrees in clinical psychology with practicums and clinical internships focused on children with special healthcare needs. One psychologist completed a pediatric pain fellowship, and one psychologist completed an autism fellowship and is clinically reliable on gold-standard diagnostic instruments for autism. Both physical and occupational therapists on our team were initially hired to work in the pediatric acute care setting and now have one day per week dedicated to the pediatric chronic pain clinic. Our doctoral-level PT completed specialized clinical training related to pediatric chronic pain, and our OT’s training background and experience uniquely include the treatment of pediatric patients with neurodevelopmental and psychiatric conditions. 

A team member from each of the four disciplines conducted an initial assessment for each new patient. Our team conducts assessments where the physician and physical therapist see one patient, while the psychologist and occupational therapist see another patient. The team briefly meets to exchange important information and swap patients. After all four disciplines have finished their initial evaluations, the team meets to review diagnostic impressions, formulate a treatment plan, and discuss the best ways to deliver the treatment plan to the patient. Patients are then provided with a half-hour feedback session with the full team similar to what is described in Schechter et al. [33]. Patients return to the clinic for full-team interdisciplinary follow-up appointments at a time interval determined by the team (typically in the range from 1 to 3 months), with more frequent services provided by specific disciplines in the interim based on recommendations (e.g., pain psychology and/or PT). Depending on availability and patient location, these weekly services are often provided by clinicians in the community, with case consultation and coordination provided by our team. At this time, our hospital does not offer intensive interdisciplinary rehabilitation services, and patients needing a higher level of care are referred to external programs.

### 2.3. Study Design

Data were gathered from a retrospective medical record review (from September 2022 to October 2023) of patients receiving interdisciplinary care through the outpatient pediatric pain clinic. Data were collected as a part of routine clinical care and were considered as exempt from informed consent procedures by the Vanderbilt University Institutional Review Board. Clinical notes from initial evaluations contained both qualitative descriptions of the patients’ experience of pain as well as quantitative measures. The autism diagnostic status was operationalized at two levels: (1) community/prior diagnosis of autism and (2) suspected autism (i.e., patients considered as very likely to meet criteria for autism). For each patient, it was also noted and further assessed by the OT if the patient had an existing or suspected diagnosis of sensory-processing disorder at the baseline.

Importantly, our clinic does not formally include resources for conducting gold-standard autism assessments or neuropsychological evaluations. Thus, “suspected” autism was based on the presence of behaviors consistent with DSM-5 diagnostic criteria both observed in the clinic and reported by parents/caregivers during a semi-structured interview focused on developmental history, with a specific focus on social affect, repetitive and restricted behaviors (including sensory interests), and reciprocal and peer interactions. 

If neurodivergence due to autism was determined to be a primary contributor to the patients’ experience of pain and functional disability, the psychologist provided a brief education to the parent/caregiver and/or patient about autism and its relevance during the initial feedback session. These patients were then referred for a formal developmental and neuropsychological evaluation. At this time, our clinic’s census does not include individuals with significant cognitive and language delays, and all the patients were verbal. 

### 2.4. Questionnaire Measures

Standardized questionnaires administered during the intake process were selected based on instruments that are commonly used in pediatric pain research and clinical settings. The Functional Disability Inventory (FDI [34,35]) parent and child report forms were used to assess limitations in physical and psychosocial functioning due to physical health from the parents’ and patients’ perspectives. The Patient Health Questionnaire-4 (PHQ-4 [36]) is an ultra-brief 4-item screener that was used to identify core depression and anxiety symptoms. The Pediatric Fear of Pain Questionnaire—Short Form (PFOPQ [37]) was used to assess patients’ pain-related fear and avoidance. Finally, the Children’s Somatization Symptom Inventory-8 (CSSI-8 [38,39,40]) was used to assess the presence and severity of a range of somatic symptoms (e.g., heart beating fast when not exercising, weakness, headaches, and abdominal pain). 

In addition to these measures, patients were asked to report their average pain intensity and were provided a checklist of words associated with affective and sensory pain qualities to help them describe their pain.

### 2.5. Summary of Clinical Observations

Over the course of the service delivery, the interdisciplinary team engaged in clinical case discussions on a weekly basis. Over time, the team identified recurrent themes related to the unique experience for providing interdisciplinary treatment for autistic youth with chronic pain. 

## 3. Results

### 3.1. Participants

Of the total of 95 patients aged 8–18 years (*M* = 14.5, *SD* = 2.77; 72% assigned female at birth, 65% identifying as female, 6% gender expansive or transgender, 28% assigned male at birth, and 33% identifying as male), 9% (*n* = 9) presented to the clinic with a prior autism diagnosis. The male-to-female sex-assigned-at-birth ratios in the autistic (66% female) and non-autistic (74% female) groups were not significantly different [*X*^2^(1, 95) = 0.38, *p* = 0.53]. 

An additional 21% (*n* = 20) of the patients were determined to exhibit clinically significant features and a developmental history consistent with autism according to expert clinician judgment, suggesting a more accurate prevalence rate of 30% (*n* = 29). Of the patients with autism, 55% (*n* = 16) presented to the clinic with widespread pain, 69% (*n* = 20) were assigned female at birth, and 17% (*n* = 5) identified as transgender or gender expansive. 

### 3.2. Questionnaire Measures

As shown in Table 1, there were no statistically significant differences, at the initial evaluation, in functional disability, anxiety and depression symptoms, fear of pain, somatic symptoms, and pain intensity between the autistic and non-autistic groups. Owing to the relatively small sample and probability of Type II errors, it is also important to consider effect sizes. The effect sizes indicated older age and greater self-reported functional disability, depressive symptoms, somatic symptom severity, and fear of pain of a small magnitude in the autistic compared to the non-autistic group. Of note, the difference in functional disability was specific to self-reports, whereas the difference based on parent reports was negligible, suggesting that the subjective experience of pain may not be readily observable or understood by parents and caregivers. Keeping in mind the subjective experience of autistic youth, it is also important to acknowledge that clinical measures have been developed and standardized in non-autistic individuals, and it is possible that adapted measures are needed to capture quantitative differences in symptom presentation and functioning. 

### 3.3. Clinical Observations and Reflections

**Figure 1:**
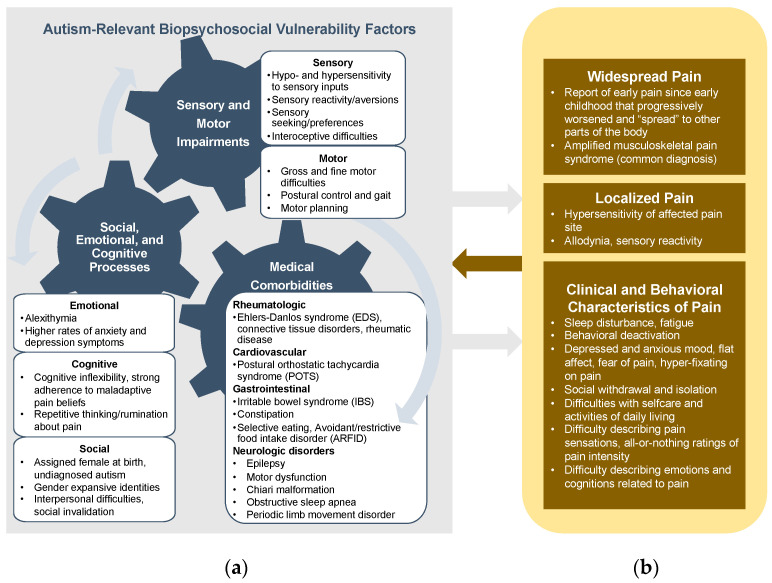
Conceptual model of (**a**) autism-relevant biopsychosocial factors that may be relevant to the development and experience of chronic pain in autistic youth; (**b**) a summary of our clinical observations of the experience of chronic pain in autistic youth.

Our team also noted qualitative differences in our clinical observations, treatment planning, and treatment delivery. We organize these observations in line with potential autism-relevant biopsychosocial vulnerability factors for pain described previously and presented in Figure 1. Specifically, sensory, motor, emotional, cognitive, and social features observed in autistic youth are summarized below. In the following sections, we describe how these biopsychosocial factors were related to our clinical observations of the individual’s experience of pain and led to qualitatively consistent adaptations or the assessment and treatment of their pain symptoms. 

### 3.4. Potential Autism-Relevant Biopsychosocial Vulnerability Factors for Chronic Pain

#### 3.4.1. Co-Occurring Medical Conditions

Consistent with the prior literature, many autistic youths in our clinic presented with co-occurring central sensitivity syndromes, including, but not limited to, chronic fatigue, fibromyalgia or amplified musculoskeletal pain syndrome, migraines, irritable bowel syndrome, and sleep disorders (e.g., restless legs syndrome). A notable subset of patients also reported a prior diagnosis of or concern for Ehlers–Danlos syndrome (EDS), a genetic connective tissue disorder associated with pain and postural orthostatic tachycardia syndrome (POTS). Notably, these diagnoses were not always medically confirmed (e.g., diagnosis by a medical geneticist for EDS and documented symptomatic orthostatic tachycardia and abnormal autonomic testing for POTS), yet patients and their families still considered these diagnostic labels to be relevant. 

Most autistic youth also reported sleep disturbances starting from a young age, including difficulty in falling asleep and staying asleep. Four of the 29 patients in the autism group reported fully nocturnal sleep routines, falling asleep between 6:00 and 9:00 a.m. and waking up between 3:00 and 5:00 p.m. Many autistic youths also presented with gastrointestinal distress (e.g., abdominal pain, chronic constipation, and nausea) and food sensitivities (often linked to sensory aversions) that led to a very limited and restricted diet.

#### 3.4.2. Sensory Processing Features

Atypical sensory seeking and avoidance behaviors were identified in all the sensory domains, including sight, sound, taste, smell, and touch. Many patients presented with a low multisensory threshold that was associated with sensory overwhelm (e.g., sensitivity to loud and unpredictable noises, crowded hallways, and bright lights), which was often described as “painful” and unbearable. In the tactile domain, a consistent pattern was the report of both a generally “high pain tolerance” (e.g., a preference for deep pressure and repetitive self-injurious behaviors, like hitting one’s head either presently or historically) and significant sensory sensitivity or aversion to light touch. These within-individual patterns of sensory dysregulation appear to contribute to increased pain severity and pain interference. Specifically, sensory sensitivities directly interfered with daily functioning and activities, including self-care and activities of daily living (e.g., hypersensitivity leading to the avoidance of taking showers because the feeling of the water touching the skin was like “being stabbed by a thousand nails”). 

Relatedly, we also noticed a pattern where autistic youth had interoceptive difficulties, such as trouble in noticing and describing internal sensory experiences. This could affect recognizing hunger, thirst, and elimination needs, which were linked to chronic pain complaints (e.g., chronic constipation associated with chronic abdominal pain and dehydration contributing to headaches). More than half of the autistic youth presented with widespread pain, which was often linked to significant fatigue, muscle weakness, and general deconditioning, with many patients reporting that they had discontinued school and daily activities or transitioned to online or homeschool programs because school was “too overwhelming” (often from a sensory perspective) and were spending most of their time in their bedrooms or in bed. 

#### 3.4.3. Motor Functioning and Primitive Reflexes

Our OT also assessed patients for primitive reflexes typically observed in infancy. Many patients had consistently retained their primitive reflexes (e.g., Moro, asymmetric tonic neck reflex, and symmetric tonic neck reflex), which typically remit in infancy. The lack of integration of these reflexes is common in individuals with neurodevelopmental disorders and is associated with difficulties with sensory processing, handwriting, midline crossing, hand–eye coordination, oculomotor deficits, reduced muscle tone, poor postural support, and attentional and working memory deficits [41]. Autistic youth tended to exhibit difficulties with postural control, gross and fine motor coordination, difficulties in grading motor forces, and difficulties with motor imitation, which were the most notable during the physical exam and when learning PT exercises. 

#### 3.4.4. Emotional Processing

Autistic patients also demonstrated differences in emotional processing and regulation compared to non-autistic patients. Many autistic youths had difficulty in describing their emotional experiences related to pain, sometimes struggling when they were given a set of emotional words to help describe their pain in the intake paperwork. Despite this, it was evident through behavioral observations, and sometimes their self-report, that they were experiencing emotional distress in response to pain (e.g., facial expressions and tension in the body). We observed that many autistic youths preferred to describe their emotional experience using metaphors or descriptors that were uniquely salient to them and their preferred interests. 

As chronic pain is a biopsychosocial process, patients were routinely screened for anxiety and depression using the PHQ-4 upon intake. Psychiatric symptoms, including anxiety, depression, and traumatic events, were then further assessed via a structured interview. We observed that many autistic youths denied depressive symptoms and focused on their emotions (e.g., depressed mood and sadness) during the clinical interview despite presenting with a flat affect and somatic–vegetative symptoms of depression (e.g., hyper- and hyposomnia, appetite changes, and feeling tired and fatigued throughout the day). In contrast, most autistic youth reported significant levels of social and generalized anxiety. Parents and caregivers often described difficulties in adjusting to unexpected changes in routines starting from early development that would lead to behavioral outbursts and significant family accommodation (i.e., accommodating the child’s scheduling preferences) to avoid behavioral outbursts and dysregulation. General emotional dysregulation and stress usually coincided with pain episodes. 

#### 3.4.5. Cognitive Processing

Autistic youth demonstrated more cognitive inflexibility and a preference for pain education to be presented in more concrete versus abstract terms. Cognitive styles were notably linear and concrete. Beliefs about pain were often in the form of all-or-nothing or polarized thinking. For example, many patients reported strong pain beliefs, e.g., that it was necessary for pain to remit before resuming functions or that there was an unidentified medical condition that was leading to chronic pain. Compared to non-autistic youth, these thoughts were less malleable and responsive to standard pain education and often hindered rapport building and treatment engagement. 

#### 3.4.6. Social Functioning and Context

Autistic youth also demonstrated social features that were relevant to their experience of pain. One of the strongest findings was the significant proportion of individuals assigned female at birth in the autism group, a condition that has been considered as more common in males. As noted earlier, 17% (*n* = 5) of the autistic youth identified as transgender or gender expansive. Most of these youth reported social stressors, including bullying and non-acceptance from peers, teachers, and family members, which contributed additional stress and led to more school avoidance. We also observed the role of social media where some patients stated that they felt that they had autism based on social media or engaging with peers who had the diagnosis. 

When asked about social functioning, many autistic youths denied feeling lonely despite having very few or no close friends, and many preferred engaging socially online (e.g., via videogames, video calls, and social media). Heightened school-related social stress was often associated with pain episodes that started upon waking, leading to school absences. 

### 3.5. Considerations for Assessment and Treatment of Chronic Pain

**Figure 2:**
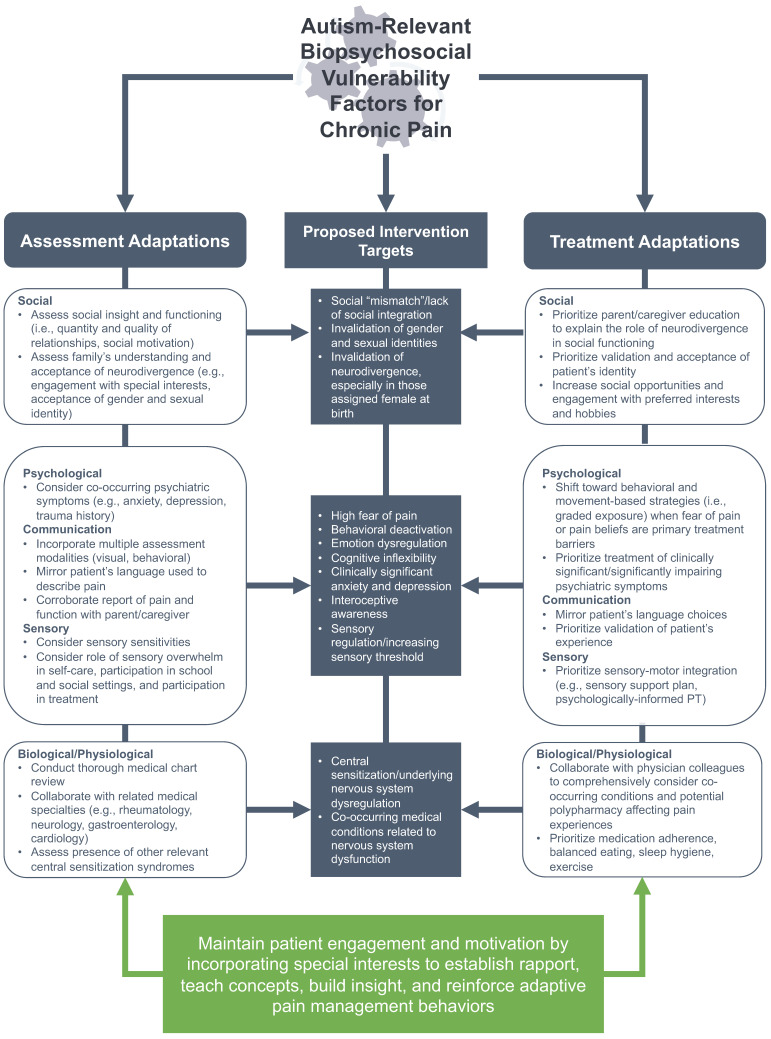
Conceptual model of how autism-relevant biopsychosocial vulnerability factors for chronic pain inform proposed intervention targets (shown in boxes in the center panel) and adaptations to assessment (left panel) and treatment (right panel). The bottom box depicts a general adaptation for incorporating the patient’s special interests to motivate and facilitate interventions.

Our reflections on the biopsychosocial presentation of chronic pain in our autistic patients allowed us to propose mechanisms and intervention targets to guide interdisciplinary treatment planning. The proposed mechanisms are summarized visually in the center panel of Figure 2 and inform adaptations for pain assessment (left panel of Figure 2) and treatment (right panel of Figure 2).

#### 3.5.1. Adaptations for Psychiatric and Medical Complexities

In the biological/physiological domain, the increased medical complexity of the autistic youth required a more thorough medical record review and collaboration with physician colleagues to consider the relevance and impact of co-occurring medical conditions in the patients’ experience of pain. The medical and psychiatric complexities of the autistic patients also increased the possibility that these patients were already prescribed medications for other conditions. For patients presenting with co-occurring psychiatric comorbidities affecting mood, serotonin and norepinephrine reuptake inhibitors (e.g., duloxetine) or tricyclic antidepressants (e.g., amitriptyline) could be considered owing to their impacts on both internalizing psychiatric symptoms and pain. In general, medications were prescribed with the goal for facilitating patients’ participation in our functional rehabilitation model, and the primary medical management of psychiatric concerns was deferred to a psychiatrist when appropriate. Autistic youth may also be using existing medications for behavioral concerns (e.g., risperidone); in these cases, our physician was cognizant of the presence of other psychoactive medications to mitigate interactions and side effects due to polypharmacy. 

#### 3.5.2. Adaptations for Pain Education and Patient–Provider Communication

A key consideration for tailoring assessment and treatment was first understanding the patient’s level of cognitive and emotional insight. If patients had difficulty in communicating and describing their pain verbally, the use of multiple modalities (e.g., verbal with visual body maps and pointing to parts of the body) was particularly relevant for information gathering to ensure that the assessment did not only rely on (incomplete) verbal reports. In these cases, it was also helpful to corroborate their report of pain with the perspectives of their parent(s) or caregiver(s), who could provide additional information regarding the patients’ pain expressions, sensory sensitivities, and development of pain throughout childhood. 

Of note, autistic patients often described pain with idiosyncratic metaphors and verbal descriptions. For example, one 14-year-old patient with chronic back pain described his chronic back pain as “like having a sword that goes straight through your body at all times”. Another 16-year-old patient with chronic and widespread migraines described his head pain as “the feeling of a mosquito bite that just never goes away”. In these instances, it was helpful to mirror and incorporate the patient’s unique descriptions of pain, even when they deviated from the language used in standard intake questions. 

When providing pain education during the initial feedback session, autistic youth benefitted from concrete descriptors and explanations of their pain condition, with simpler explanations often being more effective than more complex explanations or metaphors. It was especially effective to explain nervous system dysregulation and chronic pain in the context of their preferred interests. For example, many patients stated video games as a preferred hobby, and it was often effective to explain the central sensitization or “overprotectiveness” of the nervous system as a “software glitch” despite their “hardware” being intact (i.e., an unremarkable physical exam). 

To facilitate adherence to OT and PT exercises, patients often benefitted from the repetition of clear instructions to ensure their understanding of the postures and exercises, especially in light of their specific sensorimotor features. Difficulty in understanding the postures also led to poor adherence with their PT home exercise program and reports that they had stopped “because the exercises don’t help” or because “I *can’t* do the exercises.” In such cases, it was important to assess whether non-adherence to PT exercises was due to underlying sensorimotor challenges and to recalibrate functional targets accordingly. 

#### 3.5.3. Prioritization of Sensory Processing and Sensorimotor Integration

Baseline sensory-processing difficulties were prevalent and associated with pain, the avoidance of school and social settings, and significant behavioral deactivation. We observed that the level of the chronic and daily sensory overwhelm at the baseline needed to be addressed first before the patient could fully participate in our treatment model. Specifically, patients were equipped with a sensory support plan aimed at increasing their baseline sensory threshold to encourage general nervous system regulation and improve sensory tolerance. Sensory support plans are tailored to the individual and include exercises for increasing the patient’s sensory threshold by increasing proprioceptive input (e.g., weight-bearing exercises) and desensitization exercises involving the graded exposure to sensory sensitivities (More detailed examples of sensory support plans are available upon request). 

Sensorimotor integration was also beneficial for addressing behavioral deactivation, to the extent that the avoidance of settings was due to sensory overwhelm. Related to the baseline sensory regulation, our OT was instrumental in helping patients establish daily structures and routines to increase engagement with pleasant and distracting activities, further providing regulation to the body and nervous system at the baseline. This often involved a family therapy component where parents/caregivers would be coached to provide clear behavioral contingencies to establish daily structures and routines. 

#### 3.5.4. General Shift from Cognitive to Behavioral Strategies

As autistic individuals can exhibit more fixed, rigid cognitions and difficulties with identifying and describing their emotional experiences, a general shift from cognitive strategies to behavioral strategies was particularly effective. Specifically, a graded exposure approach was prioritized to reduce the fear of pain and help the individual “update” maladaptive pain beliefs by orienting the patient to more concrete and observable behavioral evidence while circumventing the overt discussion of emotions and cognitions. 

#### 3.5.5. Affirmation of Autistic/Neurodivergent Identities

It is notable that many of the patients suspected to meet criteria for autism in our clinic were adolescents assigned female at birth, individuals who received a “late” autism diagnosis (i.e., after 3 years old) or identified as transgender or gender expansive. These individuals often experienced nonacceptance from family members or peers and expressed feeling misunderstood by individuals in their social environment. Recent research has highlighted how autistic youth often feel pressure to “mask” or “camouflage” aspects of their autistic identity owing to the stigma of autism, social pressures, or efforts to feel a sense of belonging [42,43]. Efforts to bridge this social “mismatch” have also been associated with “autistic burnout”, a particular form of chronic stress associated with increased anxiety, depression, and suicidality, especially in autistic adulthood [44,45]. Chronic stress due to nonacceptance of neurodivergence may also be exacerbated by additional intersectionalities, such as being an autistic female or having gender expansive identities [46]. 

As underlying nervous system dysregulation is implicated in the development and maintenance of chronic pain [24], the assessment of these social stressors was critical to integrate into the conceptualization and treatment of pain in autistic youth. In the context of the treatment, it was important to maintain a treatment environment committed to the acceptance of neurodiversity and gender diversity. For family systems that were not affirming of their child, psychoeducation for parents and caregivers was prioritized to improve parent–child communication. Specifically, parent education regarding autism and parent coaching in the validation and principles of behavioral modification (i.e., reinforcing adaptive pain management behaviors) were particularly relevant. 

Relatedly, we did observe that some patients were strongly identified with medical labels or aspects of their autistic identity, as perpetuated by health-related information disseminated on social media platforms (e.g., TikTok), that were inaccurate and detrimental to their participation in our treatment model. Our team was generally aware of the self-diagnosis of either pain-related conditions or autism based on misinformation transmitted on social media. In these instances, our team was mindful to conduct a functional analysis of patients’ social media use to determine possible reasons for patients seeking medical advice from social media versus medical experts. One example of this was patients’ requests for mobility aids, even when such aids are typically inconsistent with our clinic’s functional restoration approach to treatment and unwarranted for the patients’ current symptoms based on PT, OT, and medical examinations. As social media platforms offer a sense for belonging and visibility, we conceptualized patients who were strongly identified with a disability identity or eager to acquire mobility aids as an indicator of social invalidation or a fear of invalidation. Thus, our team sought to provide affirming care by assessing and addressing potential sources of social invalidation while prioritizing psychoeducation and accurate information. 

#### 3.5.6. Maintaining Treatment Engagement and Motivation

Owing to the characteristics outlined above, our team also noticed consistent adjustments to care delivery to establish and maintain treatment engagement and motivation. Because patients are evaluated by the full team, the initial evaluation, including assessment and feedback, takes about 3–4 h. Follow-up visits have a different setup, where patients are co-treated by the physician and occupational therapist first, followed by co-treatment by the psychologist and physical therapist. This change in the clinical flow was sometimes anxiety-provoking for autistic youth who preferred to know ahead of time what the clinical flow would entail. It was also important to be mindful of the number of treaters in the room, especially when there were medical students, fellow physicians, or OT and PT students in the room. 

Finally, an important consideration unique for working with autistic youth was harnessing the power of special interests (shown at the bottom of Figure 2) to build rapport and increase or maintain treatment motivation and engagement. For example, a 10-year-old patient with chronic knee pain initially presented to our clinic without the ability to bend his right knee. He used a walker so that he did not have to put pressure on his right leg and could keep it extended. At the baseline, he was very resistant to physical touch owing to sensory sensitivity and especially could not tolerate his right knee being touched during the physical exam. He held a strong belief that there was something wrong with his knee after seeing imaging results that suggested a benign abnormality in his knee joint. He exhibited significant fear of pain but became dysregulated when asked to label his emotions associated with pain, stating, “I *hate* talking about feelings”. We built rapport by discussing his preferred interests, during which we noticed an immediate shift toward a positive affect and treatment engagement. With this patient, we created a graded exposure hierarchy based on his preferred interests, which motivated him to reach his functional goals by a certain date. For this patient, we noticed that the behavioral change was the most effective in improving his functioning and eventually shifting his pain beliefs. It was also noted that his more concrete thinking style could be leveraged to enhance his treatment. For this patient, his all-or-nothing thinking style appeared to facilitate his treatment progress; once his behaviors indicated that he *could* bend his knee (i.e., no longer “nothing”), his treatment progressed quickly toward a full recovery.

## 4. Discussion

This clinical commentary provides a review of medical records and the clinical reflections of our multidisciplinary team to provide insights into the presentation of chronic pain and potential adaptations for its assessment and treatment in autistic youth. Consistent with a growing awareness of chronic pain in autistic youth and statistics reported in other pediatric pain clinics [27], our clinical records suggest that approximately 9% of the patients had a prior diagnosis of autism but that the prevalence may have been as high as 30% based on developmental history and expert clinical judgment. The majority of the autistic individuals evaluated in our clinic were assigned female at birth, highlighting the potential importance of pain clinics as a unique treatment setting for detecting and caring for autistic females, who may be underdiagnosed and receive fewer supports compared to their male peers [47]. 

Recently published qualitative work has called for a more systematic characterization of pain among autistic youth to guide individualized treatment and improve outcomes for this underserved and vulnerable group [48]. This clinical commentary begins to address this need by integrating well-documented sensory, emotional, cognitive, motor, and social factors into the chronic-pain and autism literatures to explain high rates of chronic pain for autistic youth in a clinical setting. These factors also inform the assessment and treatment of chronic pain in autistic youth to better serve their needs. Many of our adaptations are consistent with established recommendations for adapting psychological interventions (e.g., CBT) for the treatment of anxiety and depression in autistic youth, including slowing the pace of the treatment to ensure understanding of concepts, focusing on emotional identification and regulation, using concrete and clear language, imposing structure and routine in the therapy environment, and providing sensory accommodations when needed [30]. This is a promising finding and suggests that the existing community of pain clinicians may feel empowered to use their existing skillsets effectively when treating autistic youth. 

Extending these general treatment adaptations, we also identified additional sensorimotor considerations that may be particularly unique to the emergence and treatment of chronic pain in autistic youth. Of note, the estimate of 90% of autistic youth with atypical sensory processing [7] was replicated in our clinical setting (88% in our outpatient clinic). In addition, the finding that more than half of the autistic patients in our clinic presented with widespread pain and retained primitive reflexes associated with motor impairments (e.g., difficulties with postural control and motor coordination and low muscle tone) suggests the roles of central sensitization and general nervous system dysregulation as a key intervention target that is especially relevant to autistic patients. This is consistent with established research suggesting that autistic youth are generally more stressed (i.e., dysregulated) throughout the day, as indicated by heightened diurnal cortisol levels that are exacerbated by sensory sensitivities [49]. 

Our findings are consistent with recently proposed models of pain in autism, highlighting the interplay between the molecular and neurophysiological pathways of pain processing and top-down regulatory processes that may be especially disrupted in autistic youth [50]. Early sensory dysregulation in autism may cascade to social and emotional challenges that include difficulties in orienting to and processing social and emotional information, integrating oneself into social settings, accepting and giving social touches, and developing verbal and non-verbal social communication skills [51]. Of note, “hallmark” features of autism, such as sensory and emotional dysregulation, paired with a more perseverative cognitive style and chronic stress (e.g., due to social invalidation), are factors that are understood to be associated with or lead to chronic pain in non-autistic youth. As illustrated in Figure 1, we intentionally depict autism-relevant biopsychosocial features as a system of gears that interact with and impact each other to depict how autism, when considered as a system of symptoms, may confer a uniquely greater risk for chronic pain, as it increases many biopsychosocial vulnerability factors associated with pain. Importantly, pain represents a cross-cutting feature of psychiatric (e.g., depression and anxiety) and medical (e.g., Ehlers–Danlos syndrome, amplified musculoskeletal pain, gastrointestinal disorders, and sleep disorders) conditions that are known to be heightened in autistic youth compared to the general population [52]. Thus, pediatric pain clinics may be a particularly helpful setting for addressing this cross-cutting symptom to alleviate the burden of co-occurring conditions to reduce healthcare utilization and improve the wellbeing and quality of life for autistic youth. 

Adaptations to the assessment and treatment of pain in autistic youth were the most needed when considering the combination of sensorimotor challenges, difficulties with interoception, emotional dysregulation, cognitive and behavioral inflexibility, and social challenges. Though future work is needed to develop more formal and replicable interventions and models of care, our clinical commentary highlights the importance of interventions focused on sensorimotor integration, shifting to behavioral and somatic strategies in response to inflexible pain cognitions/beliefs, and psychosocial therapies that bridge the social “mismatch” between one’s neurodivergent social identity and social contexts. To implement this, our clinic routinely incorporated a sensory support plan into treatments, with guidance from our OT to increase patients’ sensory thresholds, prioritized behavioral strategies, like graded exposure (e.g., exposure to the fear of movement and pain in a hierarchical manner), in psychological and physical therapies to change maladaptive pain beliefs, and intentionally considered the role of chronic social invalidation leading to chronic stress and pain (e.g., family therapy and parent coaching to address the chronic invalidation of neurodivergent identities). 

### 4.1. Future Directions

As sensorimotor integration is not yet routinely incorporated into pediatric chronic pain treatments, our findings will motivate future efforts to incorporate movement-based therapies and somatic experiencing into standard multidisciplinary pain treatments for autistic youth. This is also consistent with recommendations to more systematically incorporate the assessment and treatment of motor functioning to improve psychological and physical health for autistic youth [53]. Our findings also emphasize the important role of occupational therapy, a clinical specialty that is not yet routinely integrated into outpatient pediatric pain clinics, to adequately support youth with distinct sensory-processing profiles.

The high percentage of patients newly identified to meet clinical criteria for autism is also notable, especially considering that most patients had already seen many medical providers, including their primary care physician and neurology, rheumatology, orthopedics, and/or cardiology specialists, by the time they were seen in our clinic. The high rate of undiagnosed autism, especially in individuals assigned female at birth, in our tertiary pediatric pain clinic suggests that autism diagnosis may be biased toward individuals assigned male at birth, resulting in greater medical and psychiatric burdens for autistic females whose neurodivergence is not identified until they experience chronic and disabling levels of pain. As such, attention to sensorimotor features and somatic and pain symptoms may facilitate the earlier identification of autism in females to promote more timely intervention and wellbeing. 

### 4.2. Strengths and Limitations

This clinical commentary has several strengths and limitations. The wide catchment area of our hospital allowed for a representative sample of youth referred for or seeking multidisciplinary care for pediatric pain management. Despite this strength, the medical record documentation does not provide direct information about the experiences or perspectives of patients, families, or clinicians. Our patient population also comprised verbal individuals without significant cognitive and language delays, so our findings and recommendations are not applicable to the experience and treatment of pain across the full spectrum of autistic individuals, especially those who are minimally or non-verbal. Additionally, our review of the records is based on a continuous sampling of pediatric pain patients in our region and may not be generalizable to other geographical regions and cultural contexts. Further, the reflections shared were based on the clinical experiences of a limited number of practitioners, and a broader survey of clinicians who treat chronic pain in autistic youth may yield additional insights.

## 5. Conclusions

This clinical commentary of youth who sought treatment in a multidisciplinary pediatric pain clinic suggests that autism is overrepresented among youth with chronic pain and seeking treatment in a multidisciplinary outpatient setting. Our findings highlight the importance of pediatric pain settings in serving the needs of autistic youth and support the need for incorporating interventions targeting sensorimotor integration and social support to improve the deleterious effects of chronic pain in this vulnerable population. 

## Figures and Tables

**Table 1:** Means, standard deviations, and between-group differences of demographic and clinical characteristics in the Autism (i.e., prior diagnosis of autism or suspected) and No Autism (i.e., no prior diagnosis or autism features not identified in the clinic) groups. Welch’s *t*-test was used for the between-group comparison of the continuous measures. Effect sizes were estimated using Cohen’s *d* (small, medium, and large effect sizes are indicated by *d* = 0.20, *d* = 0.50, and *d* ≥ 0.80, respectively). FDI = Functional Disability Inventory [34]; PHQ-4 = 4-item Patient Health Questionnaire [36]; PFOPQ = Pediatric Fear of Pain Questionnaire [37]; CSSI-8 = 8-item Children’s Somatization Symptom Inventory [38,39,40].

Clinical Measures	No Autism (*n* = 66) *M* (*SD*)	Autism (*n* = 29) *M* (*SD*)	*t*-Test	*p*-Value	Cohen’s *d*
Age (years)	14.2 (2.82)	15.2 (2.55)	*t*(58.76) = −1.69	0.10	−0.37
Functional Disability (FDI)—Patient Report	21.4 (10.5)	25.9 (10.7)	*t*(36.78) = −1.70	0.10	−0.43
Functional Disability (FDI)—Parent Report	23.26 (12.17)	23.25 (11.18)	*t*(56.80) = 0.005	0.99	0.001
Anxiety Symptoms (PHQ-4)	2.67 (1.95)	2.89 (2.06)	*t*(46.61) = −0.47	0.64	−0.11
Depressive Symptoms (PHQ-4)	1.48 (1.78)	2.04 (1.68)	*t*(51.86) = −1.41	0.16	−0.32
Fear of Pain (PFOPQ)	20.9 (8.11)	22.8 (5.73)	*t*(68.09) = −1.25	0.21	−0.21
Somatic Symptom Severity (CSSI-8)	13.7 (6.91)	15.6 (6.56)	*t*(51.02) = −1.26	0.22	−0.28
Pain Intensity at Evaluation	4.27 (2.78)	4.69 (2.16)	*t*(68.09) = −0.79	0.43	−0.17

## Data Availability

The quantitative data presented in this study are available on request from the corresponding author. The data are not publicly available owing to patient confidentiality.
